# Multidisciplinary teams for cardiogenic shock

**DOI:** 10.18632/aging.102104

**Published:** 2019-07-17

**Authors:** Saarwaani Vallabhajosyula, Gregory W. Barsness, Saraschandra Vallabhajosyula

**Affiliations:** 1 Department of Cardiovascular Medicine, Mayo Clinic, Rochester, MN 55905, USA; 2 Division of Pulmonary and Critical Care Medicine, Department of Medicine, Mayo Clinic, Rochester, MN 55905, USA

**Keywords:** acute myocardial infarction, cardiogenic shock, shock team, multidisciplinary approach, team-based approach, cardiac intensive care

**Figure 1:**
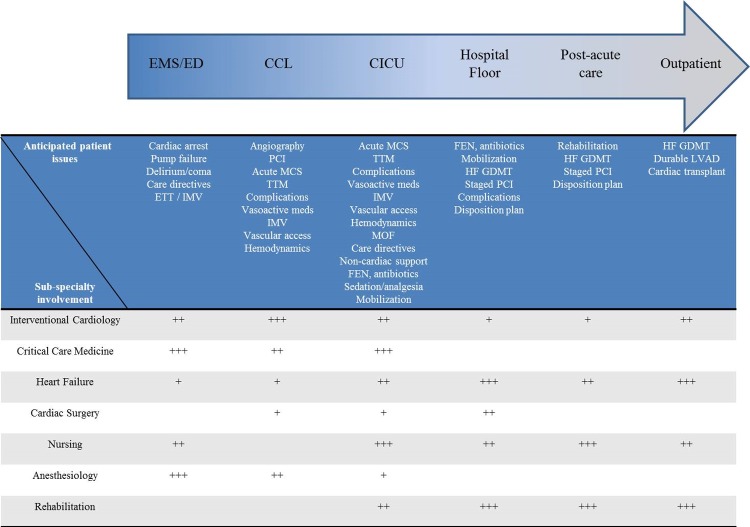
**Trajectory of AMI-CS highlighting the role of multidisciplinary team members.** The arrow from left to right shows the anticipated trajectory of the evolution of AMI-CS classified by geographic locations within the hospital. These are only hypothetical scenarios and may differ from real patient situations. The blue row on the table lists the common issues anticipated in these geographic locations. The grey rows on the table list the various multi-disciplinary team members and their extent of involvement (+ is low, ++ is moderate, +++ is high) in each of these areas.

Cardiogenic shock (CS) is a state of end-organ hypoperfusion due to a primary cardiac problem associated with high mortality and morbidity in the contemporary era [1,2]. More than 80% of all CS cases in the modern era are due to acute myocardial infarction (AMI) [1,2]. Despite rapid advancements in the field of interventional cardiology and percutaneous coronary interventions (PCI), AMI-CS continues to be associated with high mortality in excess of 30-40% [3-5]. However, despite shorter door-to-balloon times and greater use of coronary angiography and PCI in recent years, there appears to be minimal incremental mortality benefit in this population [1]. AMI-CS follows a ‘hemo-metabolic’ cascade, wherein the initial hemodynamic insult subsequently results in metabolic derangement causing multi organ failure [1,6]. Our group has previously shown that patients with AMI-CS have developed greater rates of non-cardiac organ failure highlighting the need for holistic management of these patients [1]. In light of these considerations, there is a crucial need for developing paradigms for multi-disciplinary care for AMI-CS patients (Figure 1) [5,7,8]. Patients with AMI-CS often have a dynamic pathophysiology and a rapidly evolving clinical picture as noted in our Figure 1. Care for such patients frequently involves anticipating patient trajectories, closely evaluating for complications, management of complications, and preventing/treating non-cardiac organ failure. Assessment of the hemodynamic profile, evaluating coronary disease and rapid revascularization form the cornerstone of therapy for these patients [3,4,6,8]. Not infrequently, these patients developed concomitant cardiac arrest further worsening their CS [1]. Collaborative care between the interventional cardiologist and critical care cardiologist is crucial for developing strategies related to coronary revascularization, acute mechanical circulatory support (MCS), management of vasoactive infusions, use of targeted temperature management, and prevention of non-cardiac organ failure [4]. During the cardiac intensive care unit stay, these patients typically need close management of vasoactive infusions, evaluation of ongoing MCS, placement of new or more potent MCS, management of multi organ failure and planning for exit strategies [1,4]. After acute stabilization, these patients need close management of heart failure, rehabilitation and advanced planning towards recovery of ventricular function or durable ventricular support [4,6].

Therefore, given the acuity of this patient population, and the need for complex decision-making, there is an imminent need for a multidisciplinary team-based approach, i.e. a ‘Shock Team’ [5,7,8]. These teams often include specialists from interventional cardiology, critical care medicine, heart failure, anesthesiology, cardiothoracic surgery, perfusion services, nursing and rehabilitation [4,5,7,8]. Institution-level data from across the United States have shown promising results with these multi-disciplinary care models [3-5,7,8]. The Detroit Cardiogenic Shock Initiative (CSI) and now National CSI adopted a model of early hemodynamic assessment with liberal use of a percutaneous left ventricular assist device (pLVAD) for achieving hemodynamic stability [3,4]. In their single-arm multi-center study, Basir et al. showed a survival to discharge of 72% and identified predictors of poor outcomes in this acutely ill population [3]. In a quality improvement initiative, Tehrani et al. used a before-after model to evaluate their outcomes with a standardized team-based approach to management of AMI-CS [5]. They noted a hospital survivorship of 58% and 77% in 2017 and 2018 compared to lower rates in 2016. Furthermore, they confirmed findings from the National CSI study and identified predictors of poor outcomes including but not limited to end-organ failure, advanced age, elevated lactate and decreased cardiac power output [5]. In a single center study using a standardized algorithm, Garan et al. reported similar outcomes with a pLVAD and veno-arterial extracorporeal membrane oxygenation (VA-ECMO) in patients with AMI-CS [8]. In this study, the presented a detailed algorithmic approach to selection of MCS which place a central role in the management of AMI-CS. However, it is important to note that unlike the intra-aortic balloon pump, the pLVAD and VA-ECMO have not been evaluated comprehensively in randomized trials powered appropriately towards hard outcomes. The ongoing DanGer Shock trial will evaluate the pLVAD to current standards of care, the results of which will likely influence the decision-making in AMI-CS patients. Our prior work using a large nationally representative database, has shown significant hospital-level disparities, that cannot be fully accounted for by patient factors or acuity of illness, suggesting heterogeneity in the management of these patients [2]. In light of these findings, there have been recent opinion pieces and societal statements that have called for regionalization of AMI-CS care [4,6]. Lastly, it is important to recognize that AMI-CS is a spectrum and has an anticipated trajectory (Figure 1). However, these patients frequently developed complications related to coronary, vascular, organ failure and arrhythmic issues that deviate the patient from the anticipated clinical trajectory. Furthermore, not all CS are created equal and therefore these patients need close and repeated evaluations during their hospital stay to identify triggers, etiology, deterioration and recovery.

In conclusion, the development of standardized processes of care and multi-disciplinary care teams is the first step towards ensuring safe and optimal patient care for this acutely ill population. Dedicated clinical research into pathophysiology and disease-specific factors in AMI-CS is warranted in an attempt to improve clinical care and outcomes.
